# *RAX* and anophthalmia in humans: Evidence of brain anomalies

**Published:** 2012-06-02

**Authors:** Hana Abouzeid, Mohamed A. Youssef, Nader Bayoumi, Nihal ElShakankiri, Iman Marzouk, Philippe Hauser, Daniel F. Schorderet

**Affiliations:** 1 Jules Gonin Eye Hospital, Lausanne, University of Lausanne, Lausanne, Switzerland; 2 Department of Radiology, CHUV, Lausanne, University of Lausanne, Lausanne, Switzerland; 3 Genetics Unit, Department of Pediatrics, University of Alexandria, Alexandria, Egypt; 4 Department of Ophthalmology, University of Alexandria, Alexandria, Egypt; 5 IRO - Institute for Research in Ophthalmology, Sion, Switzerland; 6 EPFL - Ecole polytechnique fédérale de Lausanne, Switzerland Faculty of Life Sciences, Lausanne, Switzerland

## Abstract

**Purpose:**

To report the clinical and genetic study of two families of Egyptian origin with clinical anophthalmia. To further determine the role of the retina and anterior neural fold homeobox gene (*RAX*) in anophthalmia and associated cerebral malformations.

**Methods:**

Three patients with clinical anophthalmia and first-degree relatives from two consanguineous families of Egyptian origin underwent full ophthalmologic, general and neurologic examination, and blood tests. Cerebral magnetic resonance imaging (MRI) was performed in the index cases of both families. Genomic DNA was prepared from venous leukocytes, and direct sequencing of all the exons and intron-exon junctions of *RAX* was performed after PCR amplification.

**Results:**

Clinical bilateral anophthalmia was observed in all three patients. General and neurologic examinations were normal; obesity and delay in psychomotor development were observed in the isolated case. Orbital MRI showed a hypoplastic orbit with present but rudimentary extraocular muscles and normal lacrimal glands. Cerebral MRI showed agenesis of the optic nerves, optic tracts, and optic chiasma. In the index case of family A, the absence of the frontal and sphenoidal sinuses was also noted. In the index case of family B, only the sphenoidal sinus was absent, and there was significant cortical atrophy. The three patients carried a novel homozygous c.543+3A>G mutation (IVS2+3A>G) in *RAX*. Parents were healthy heterozygous carriers. No mutations were detected in orthodenticle homeobox 2 (*OTX2*), ventral anterior homeobox 1 (*VAX1*), or sex determining region Y-box 2 (*SOX2*).

**Conclusions:**

This is the first report of a homozygous splicing *RAX* mutation associated with autosomal recessive bilateral anophthalmia. To our knowledge, only two isolated cases of anophthalmia, three null and one missense case affecting nuclear localization or the DNA-binding homeodomain, have been found to be caused by compound heterozygote *RAX* mutations. A novel missense *RAX* mutation was identified in three patients with bilateral anophthalmia and a distinct systemic and neurologic phenotype. The mutation potentially affects splicing of the last exon and is thought to result in a protein that has an aberrant homeodomain and no paired-tail domain. Functional consequences of this change still need to be characterized.

## Introduction

Anophthalmia (absence of the eye) is rare and the most severe ocular dysgenesis. Often discussed together with microphthalmia (small eye), anophthalmia and microphthalmia have a cumulative approximate frequency of one to two in 10,000 births [1-3]. Despite this low rate, the severity of the disease and the role it can play in understanding normal eye development justify thorough study of human anophthalmia.

Several anophthalmia- or microphthalmia-causing genes have been identified to date and include, among others, paired box 6 (*PAX6*), orthodenticle homeobox 2 (*OTX2*), sex determining region Y-box 2 (*SOX2*), visual system homeobox 2 (*VSX2*), ventral anterior homeobox 1 (*VAX1*), and retina and anterior neural fold homeobox (*RAX*) [4-6]. *SOX2* is to date the gene most frequently involved in anophthalmia and accounts for up to 10% of cases [5,7].

*RAX* is a homeobox gene that plays a major role in human and vertebrate eye development. In mice, *Rax* is involved in optic vesicle formation, and loss of function is responsible for anophthalmia and leads to brain malformation [8]. Expressed early in retinal development, *RAX* is thought to determine the fate and proliferation of retinal cells [9]. Few reports of *RAX* mutations in humans have been published, and these include anophthalmia, microphthalmia, and eye coloboma [10,11].

## Methods

### Patients

**Figure 1:**
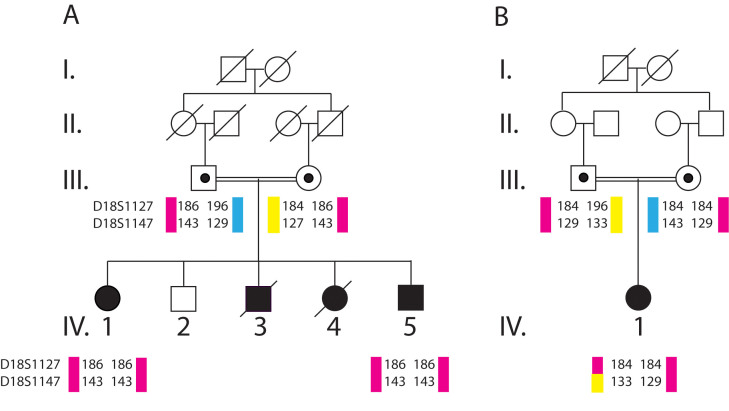
Pedigrees of the two Egyptian consanguineous families with autosomal recessive anophthalmia. Squares and circles represent men and women, respectively. Darkened symbols denote affected family members. A double line indicates consanguinity.

This study was approved by the Ethics Committee of the Faculty of Medicine of the University of Alexandria, Egypt, and was conducted in accordance with the tenets of the Declaration of Helsinki. Written informed consent was obtained from each participant or parent. Three patients with anophthalmia belonging to two consanguineous families and their first-degree relatives were included in this study (Figure 1: family A, patients IV:1 and IV:5; family B, the affected daughter). Parents of the affected patients were first-degree cousins in both families. In family A (Figure 1), two affected children died in infancy because of severe dehydration caused by diarrhea (IV:3, IV:4). The two families were from the District of Alexandria in northwestern Egypt. All available subjects underwent full ophthalmic, general, and neurologic examinations at the Departments of Ophthalmology, Pediatrics, and Neurology of the University of Alexandria, Egypt, respectively. Special attention was paid to assessing the presence of associated anomalies such as mental retardation. A cerebral magnetic resonance imaging (MRI) was performed on both index patients (patient IV:5 of family A and the affected daughter of family B; Figure 1). Magnetic resonance imaging (MRI) were reviewed by two different neuroradiologists. MRI acquisition techniques included conventional T1- and T2-weighted multisection images (5 mm slice) on a 1.5-Tesla MR imaging system (Siemens, Helsinki, Finland).

### Molecular analysis

Microsatellite markers flanking all known genes associated with micro/anophthalmia were evaluated for homozygosity in families A and B. DNA from patients was extracted from peripheral leucocytes as previously described [12]. Direct bidirectional sequencing of all PCR-amplified exons including the 3′- and 5′ untranslated regions as well as the adjacent intron junctions was performed with the ABI Dye Terminator (Applied Biosystems, Zug, Switzerland), version 1, and electrophoresed on a 3130XL ABI Genetic Analyzer (Applied Biosystems). Primers were obtained with the help of the Primer3 program and are listed in Table 1. Sequences were compared to the reference sequence NM_000280.3 using Chromas version 2.23 (Technelysium, Tewantin, Australia). The adenine of the ATG translation start site was set to one. The heterozygous mutation identified with direct double-strand sequencing was further validated with denaturing high-performance liquid chromatography (DHPLC) on a WAVE system (TEAA; Transgenomics, Crewe, England) with the temperature set to 64.6 °C and the start buffer B to 58%. The same DHPLC approach was used to check for the presence of the sequence variant in the DNA of 80 ethnically matched and 96 European control individuals. The role of the variant in splicing was evaluated with in silico analysis using the SplicePort computer program [13], and DNA sequences of various species encompassing the variant were aligned using options available through Ensembl.

**Table 1:** Primers used for direct sequencing of *RAX*.

**Exon**	**Forward primer (5′-3′)**	**Reverse primer (5′-3′)**	**Size of PCR product**
1	GCCTCTCCTCTCCGTCTCC	GGGCGCCCGAACGGCCTC	380 bp
2	GGAGTGCATCTGACCCTCC	TGGCTGCAATTTGGGCCTCG	351 bp
3a	GAGCTGAACCGGCTCAGG	GGATCCCAAGACGTTCCCC	602 bp
3b	AAGTTCCCGCTGGACGAG	CAGAGTCGAAACAAAACAAGCA	597 bp
3c	GGAGACCCCCAGATAACCAT	CGGAGATCTGCTTGGTGAAC	567 bp
3d	CAGCGGCAGCTGATATTTTC	GGCATCCAGGTTCTGCTG	554 bp
3e	CTAGGGCGAGGAAGGAATCT	TTAGACCCGCAGAGAAAGGA	557 bp
3f	TAATCATCGTCCCCATTTCC	ACTTGGAGCCCATGAAGTTG	453 bp

Exon 3 was split in 6 overlapping DNA fragments. bp: base pairs

## Results

### Clinical findings

**Figure 2:**
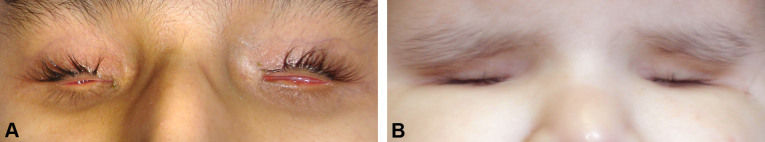
Facial features of the anophthalmic patients. **A**: Family A: Patient IV:1 at 16 years of age. Note the complete absence of eyes and the large eyebrows with low insertion on the superior eyelid. The patient presented normal psychomotor development. She had a high arched palate and was normal in height, weight, and head circumference. **B**: Family B: the affected daughter at 7 months of age. As in family A, note the large eyebrows with low insertion on the superior eyelid. Global delay in development, obesity (weight: 7 SD above mean for age and sex, height: 90th percentile), abnormal head circumference (7th percentile), and a high arched palate were observed in this patient. On the genetic level, both patients carried an IVS2 + 3A>G homozygous *RAX* mutation.

Clinical bilateral anophthalmia was observed in all three affected children of the two families. In family A, both affected children, patients IV:1 and IV:5, had complete absence of eyes, large eyebrows, and a low insertion on the superior eyelid (Figure 2). They had a high arched palate and were normal in height, weight, and head circumference. Both presented normal psychomotor development; general and neurologic examinations were normal for all family members.

As in family A, the affected daughter of family B had large, flaring eyebrows and a low insertion on the superior eyelid (Figure 2). This patient had global development delay, obesity (weight: +7 standard deviation [SD] for age and sex, height: 90th percentile), abnormal head circumference (7th percentile), and a high arched palate.

### Magnetic resonance imaging findings

**Figure 3:**
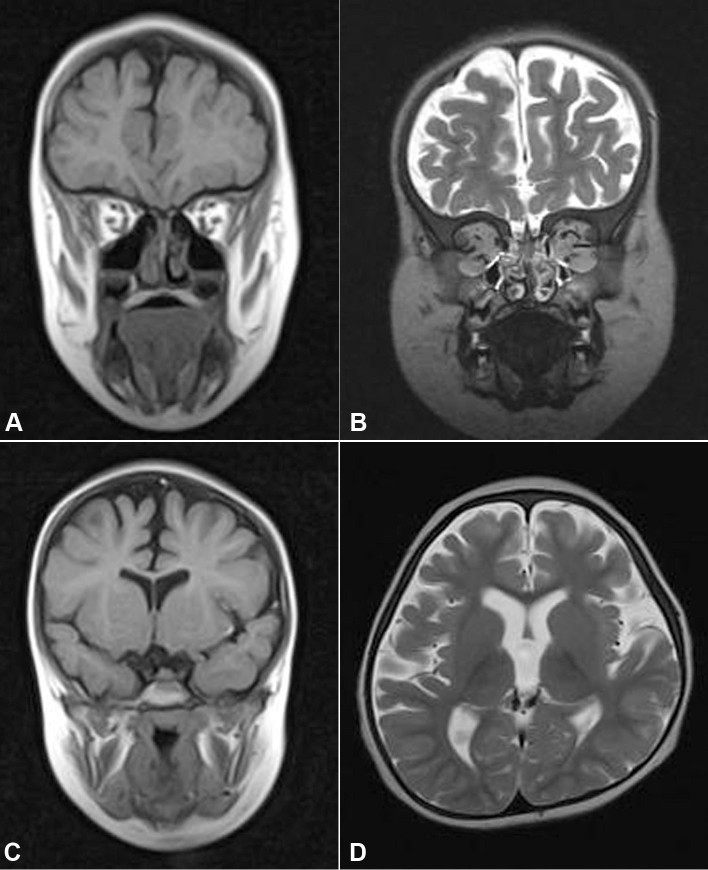
Cerebral and orbital MRI images. **A**: Family A, Patient IV:5. **B**: Family B, affected daughter. Orbital hypoplasia. Coronal images, T2-weighted. Agenesis of the eye globes. Rudimentary extraocular muscles. Present and normal lacrimal glands. **C**: Axial image. T1-weighted. Family A, patient IV:5. Absent frontal sinus. **D**: Axial image. T2-weighted. Family B, affected daughter. Corticosubcortical atrophy, predominantly in the frontotemporal lobes, with ex vacuo dilation of the ventricles.

In patient IV:5 (Figure 3A,C) of family A and the affected daughter (Figure 3B,D) of family B, orbital MRI showed a hypoplastic orbit with present but rudimentary extraocular muscles and a normal lacrimal gland. A cerebral MRI showed agenesis of the optic nerves, optic tracts, and optic chiasma, and a normal pituitary gland. In patient IV:5 (family A), the absence of frontal and sphenoidal sinuses was also noted. In the affected daughter (family B), only the sphenoidal sinus was absent, and significant corticosubcortical atrophy, predominantly in the frontotemporal lobes, was observed with ex vacuo dilation of the ventricles.

### Molecular findings

**Figure 4:**
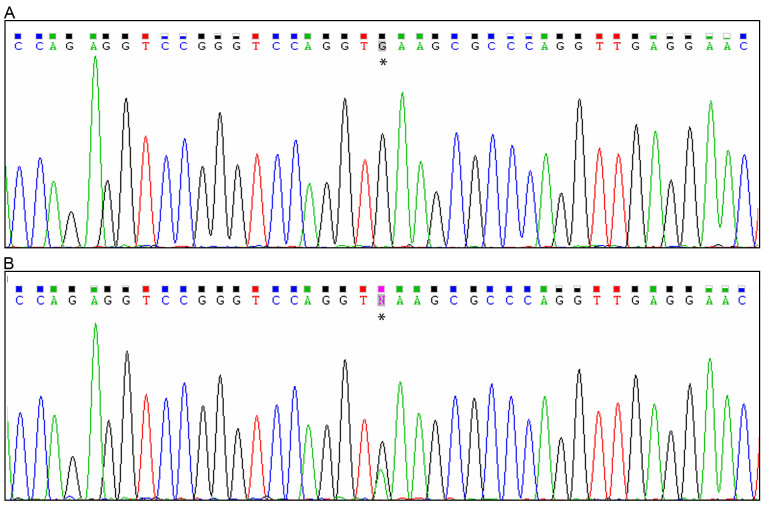
Electropherograms of *RAX* mutations. Asterisks denotes mutated bases. **A**: Electropherogram of patient IV:1 (family A) showing a homozygous c. 543+3A>G *RAX* mutation. **B**: Electropherogram of patient IV:1’s father (family A) showing the heterozygous mutation.

**Figure 5:**
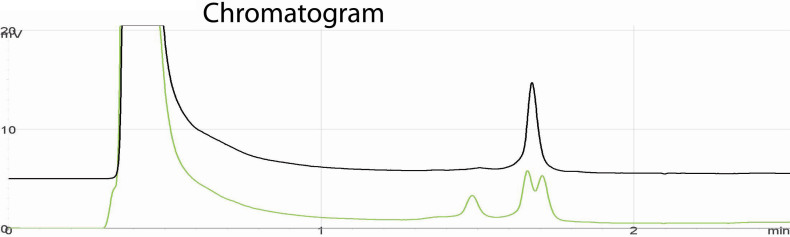
Chromatogram obtained on a Wave DHPLC system. Upperlane: PCR product of RAX exon 2 in a normal individual (family A:IV:2) showing one single peak, indicative of a unique product. Lowerlane: PCR product of the same region in an heterozygous individual (family A, father of IV:1). Three peaks corresponding to various heterodimers are visible. These heterodimers are obtained from different arrangements between wild-type and mutated DNA strands. y-axis: intensity, x-axis: retention time. The difference in the shape of the curves between normal and heterozygous individuals allows for an easy estimate of the frequency of the mutation in normal individuals.

Molecular analysis with markers D18S1127 and D18S1147, two markers flanking *RAX*, showed a homozygous haplotype in the affected individuals of family A suggesting that *RAX* could be involved in the disease. Sequencing of all the exons and 3′ and 5′ UTR identified a c.543+3A>G mutation (IVS2+3A>G) in the splice donor of the second intron of *RAX* (Figure 4A). All three affected individuals from both families were homozygous for the mutation while their healthy parents were heterozygous (Figure 4B). The mutation was absent in the healthy sibling (IV:2) in family A (Figure 5). The mutation was not detected in 80 ethnically matched healthy individuals (50 Algerians and 30 Egyptians) and in 96 European controls, and has not been previously reported. No other mutations were detected in *OTX2*, *VAX1*, or *SOX2*.

The third base of the donor site is conserved in various mammals as well as in chickens (*Gallus gallus*) and fugu fish (*Takifugu rubripes*). The donor site, however, is no longer conserved in zebrafish and more distantly related species such as *Caenorhabditis elegans* (Table 2) [13]. In silico analysis of the splice potential donor site of the wild-type sequence of the second intron using the computer program SplicePort gave a score threshold of 1.64346 with a predicted sensitivity of 88.8%, a score usually associated with good splicing potential. This score was reduced to 0.39398 when the IVS2+3A>G variant was evaluated, a score much lower but not indicative of a nonfunctional donor. Haplotype analysis was also performed in family B. The alleles for both microsatellites were different from family A. Based on the analysis of the three individuals available, it seems that a recombination occurred between D18S1127 and D18S1147 in the affected child. No single nucleotide polymorphisms (SNPs) were identified in any of the sequences performed in the three affected individuals.

**Table 2:** Alignments of various exon 2–intron 2 sequences.

**Species**	**Ensembl ID**	**Exon/Intron boundary**
*H. sapiens*	ENSG00000134438	R V Q
		cgc gtc cag gta**a**agcgc
*P. troglodytes*	ENSPTRG00000010058	..G ... ... .........
*B. Taurus*	ENSBTAG00000022826	..A ... ... ........t
*O. cuniculus*	ENSOCUG00000010455	... ..G ... ....g....
*F. catus*	ENSFCAG00000006503	..G ... ... .........
*R. norvegicus*	ENSRNOG00000016944	..G ... ... ....gctct
*M. musculus*	ENSMUSG00000024518	..G ... ... ....gtaag
*G. gallus*	ENSGALG00000013431	... ..G ... ...gg.atg
*T. rubripes*	ENSTRUG00000010917	..A ..G ... ...tgtgcg
*O. latipes*	ENSORLG00000006024	... ..G ... ..g.gtgct
*X. tropicalis*	ENSXETG00000021154	A.G ..G ... ..g.gt.at
*D. rerio*	ENSDARG00000071684	..A ..G ... ..gtgtatt
*C. elegans*	ZK265.4	..A ..T ... ..g.ga.at

Identical bases at the exon 2 – intron 2 boundary are replaced with a dot. The RVQ amino acids are conserved in all the described species. The “a” in bold in *H. sapiens* intron corresponds to the (IVS2+3A>G) mutation.

## Discussion

This is the first report of human bilateral anophthalmia caused by a homozygous mutation of *RAX*. The identified c.543+3A>G (IVS2+3A>G) splice mutation has not been reported before. Cosegregation analysis in family A confirmed the previously suggested recessive inheritance of *RAX* mutations in human anophthalmia [10,11]. The mutation affects splicing of the last exon as shown by the strong reduction in splicing potential as determined by SplicePort in silico analysis. This could potentially generate a truncated protein. *RAX* mutations are rare, and it seems unlikely that two unrelated families would share a similar mutation unless they share a common DNA haplotype. To identify this DNA segment, we performed a haplotype analysis in family B using the same microsatellites flanking *RAX*. This analysis showed that the three affected individuals do not share the DNA segment defined by the microsatellites used. A smaller common region cannot be excluded and, based on the low frequency of the reported *RAX* mutations, seems likely. Interestingly, no SNPs were observed in the sequences of the three exons and exon-flanking intronic regions.

Only four reports of *RAX* mutations in ocular dysgenesis are present in the literature (see Table 3). Voronina et al. [10] reported a child with unilateral partial anophthalmia (presence of remnants of the globe) due to a compound heterozygous *RAX* mutation, one missense and one nonsense. Microphthalmia and sclerocornea with persistent fetal vasculature and retinal detachment were present in the other eye. The mother was a healthy carrier of one mutation, and autosomal recessive inheritance was thus suggested. The child was autistic, but no cerebral malformations were observed on the MRI. The second report describes a child with complete bilateral anophthalmia (no remnants of the globe) and normal development and cerebral MRI except for the absence of optic nerves and hypoplastic chiasma. Again, compound heterozygosity was reported but with two nonsense mutations. The parents of the child were not tested for *RAX* mutations.

**Table 3:** Reported Mutations in the *RAX* gene and ocular phenotypes.

***RAX* Mutation**	**Ocular phenotype RE/LE**	**Cerebral malformations**	**Affected patients**	**Patient origin**	**Parent *RAX* mutation**	**Reference**
p.Q147X/p.R192Q Compound heterozygous	Anophthalmia/Sclerocornea, persistent fetal vasculature, retinal detachment.	None (MRI)	1	Not reported	Healthy mother, heterozygous carrier R192Q	[10]
p.Ser222ArgfsX62/p.Tyr303X Compound heterozygous	Anophthalmia/ Anophthalmia	Hypoplastic optic tracts and chiasm, normal brain. (on MRI)	1	Algeria	Not tested	[11]
Heterozygous p.R66T	Chorioretinal coloboma/Normal	Not reported.	1	Not reported	Not tested	[15]
Heterozygous p.T50P	Microphthalmia/Normal	Septum pellucidum agenesis, cortical atrophy, LE optic nerve atrophy (on CT scan)	1	Mexico	Not tested	[14]
Heterozygous p.R110G	Anophthalmia/Normal	Hydrocephalus (on CT scan)	1	Mexico	Not tested	[14]
Homozygous p.IVS2+3G>A	Anophthalmia/ Anophthalmia	Agenesis of the ONs, tracts and chiasm. (on MRI)	2	Egypt	Father and mother, heterozygous healthy carriers	Abouzeid et al. (present study)
Homozygous p.IVS2+3G>A	Anophthalmia/ Anophthalmia	Agenesis of the ONs, tracts and chiasm. Significant cortical atrophy. (on MRI)	1	Egypt	Father and mother, heterozygous healthy carriers	Abouzeid et al. (present study)

ON: optic nerve.

Our study confirms the autosomal recessive inheritance of some cases of human anophthalmia, and it seems from the few available cases that two null alleles are necessary to induce such a severe phenotype as anophthalmia. This is suggested by the presence of a healthy mother and a maternal grandmother in the first report [10] and confirmed by the presence of the four parents of this report who were all heterozygous carriers with normal eye globes. Our report is at odds with a recent report by Gonzales-Rodriguez et al. [14] in which two patients affected with unilateral anophthalmia and unilateral microphthalmia, respectively, as well as cerebral malformations, were reported to carry a single heterozygous *RAX* mutation. Neither mutation was found in 400 normal controls suggesting their pathogenicity although the missense mutation carried by the patient with microphthalmia was found to be non-deleterious by the algorithm used by the authors to predict functional impact. The authors concluded that a single *RAX* allele mutation could cause the presented phenotype. Nevertheless, since we did not perform segregation or functional analysis in this study, the possibility that the second allele carries an undiscovered mutation cannot be ruled out. The fact that the patients reported by Gonzalez-Rodriguez et al. [14] had a severe phenotype, which includes cerebral malformations, while we report healthy carriers of potentially truncating mutations might support our hypothesis. Moreover, a recent publication on heterozygous *RAX* mutation associated with ocular dysgenesis described a child with ocular coloboma only and no other associated malformations [15], suggesting again the mild or normal ocular phenotype that heterozygous *RAX* mutation carriers can harbor. As a matter of fact, mice heterozygous for the *Rax* gene mutation develop normal eyes as well [8].

Although few reports are available, the type of *RAX* mutation might be determinant for the ocular phenotype. The presence of *RAX* nonsense mutations is associated with anophthalmia in the reports of Voronina et al. [10], Lequeux et al. [11], and Gonzalez-Rodrigues et al. [14] and this present study. Microphthalmia [14] and coloboma [15] are associated with *RAX* missense mutations. Further studies including functional analyses are required to corroborate this hypothesis.

This is the first report of severe cerebral malformation observed on cerebral MRI associated with homozygous *RAX* mutations in humans. In addition to the absence of optic nerves, chiasm and optic tracts, which was also reported, albeit in a milder form, by Lequeux et al. [11], patient IV:5 of family A presented a significant corticosubcortical atrophy with ex vacuo dilation of the ventricles. *Rax* has been shown to play a major role in brain development in different animal models including mice [8,16]. This observation is thus not surprising and may represent another expression of the severity of the phenotype. Because no cerebral malformation was observed in the case studied by Voronina et al. [10], these authors attributed a smaller role to *RAX* in the development of the ventral forebrain than to the morphogenesis of the eye. Our report challenges this conclusion; the type of mutation may instead be incriminated by the presence of cerebral malformations. Further observations, however, are necessary to draw any firm conclusions.
